# Laryngeal hemangioma

**DOI:** 10.1016/S1808-8694(15)31009-0

**Published:** 2015-10-19

**Authors:** Regina Helena Garcia Martins, Arlindo Cardoso Lima Neto, Graziela Semenzate, Renan Lapate

**Affiliations:** a Assistant Professor, PhD in Surgery - Medical School of Botucatu - Unesp. Head of the Voice and Speech Sciences Department - Professor of Otorhinolaryngology - Paulista State University - Unesp, Campus de Botucatu; b MD. ENT Resident - Otorhinolaryngology - Medical School of Botucatu - UNESP; c MD. ENT Resident - Otorhinolaryngology - Medical School of Botucatu - UNESP; d Medical Student - Medical School of Botucatu - UNESP; e Department of Otorhinolaryngology - Medical School of Botucatu (UNESP)

**Keywords:** dysphonia, hemangioma, larynx

## INTRODUCTION

The hemangioma is the most common vascular tumor, involving the head and neck in 60% of cases. It is rare in the larynx. In children hemangiomas are more frequent on the subglottis where it causes stridor and dyspnea^1^, ^2^. In adults the most frequent site for hemangiomas is the supraglottis, and symptoms may be absent or restricted to mild forms of dysphonia or dysphagia. Frequently the supraglottic hemangioma is an endoscopic finding^3^, ^4^.

## PRESENTATION OF CASES

Patients in this study were seen at the Clinical Hospital of the Botucatu Medical College (Unesp).

### Case 1

**Figure 1:**
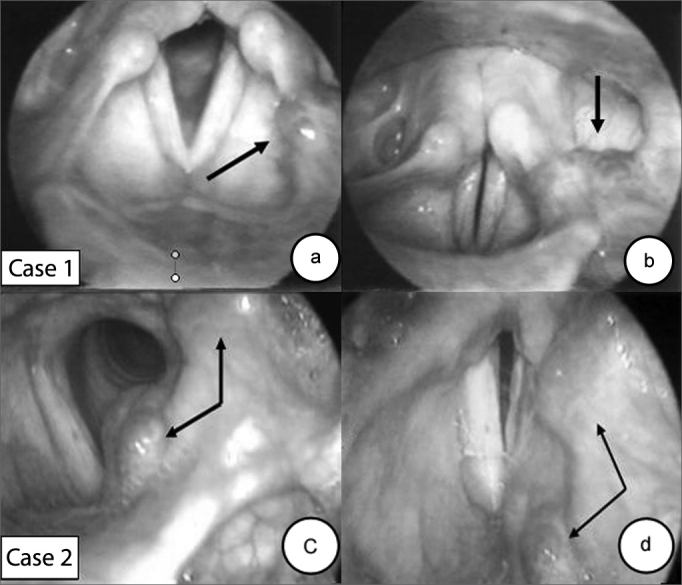
Laryngeal hemangioma (arrows).

This was a 29-year-old female patient reporting hoarseness since childhood. She had a rough, low, breathy and dyplophonic voice. Rigid telescopy revealed a 2.5 cm violet sessile tumor on the left aryepiglottic fold, atrophic vocal folds and a fusiform slit on phonation. (Figures 1a and b).

### Case 2

This was a 34-year-old male patient reporting hoarseness under conditions of vocal abuse for the past five years. In the past month, during upper digestive tract endoscopy, a “bluish spot on the larynx” was seen. Rigid telescopy revealed a bluish sessile tumor involving the left vestibular fold, the vocal fold and the base of the tongue. The left vocal fold was irregular and atrophic (Figures 1c and d).

In both cases patients did not report respiratory symptoms. Clinical monitoring was the chosen option for these cases.

## DISCUSSION

When asymptomatic, laryngeal hemangiomas may be diagnosed by other specialists, such as during endoscopy, as in case 2. The patient had no significant vocal symptoms in his daily activities.

Vocal folds were altered in both cases. They were atrophic in case 1, with a reduced glottic wave and a fusiform slit, which suggest a probable vocal cord sulcus. Pontes et al.^5^, in describing minimal structural injuries, underlined the possible association between these injuries in the same patient, due to the embryogenic peculiarities of the larynx. In this case, confirmation of the diagnosis could be reached by direct laryngoscopy, a procedure that the patient rejected.

Endoscopy is almost always sufficient for the diagnosis of a hemangioma. Other exams, such as magnetic resonance imaging with contrast and angiography, are reserved for large tumors and for surgical patients with respiratory symptoms. Biopsies are not indicated due to the risk of severe bleeding^2^, ^3^, ^4^, ^6^.

## FINAL COMMENTS

The laryngeal hemangioma may or not cause symptoms. Endoscopy should be careful and detailed due to possible extension of the lesion to adjacent structures and associations with other laryngeal lesions.
